# Characterization of a functional C3A liver spheroid model†
†Electronic supplementary information (ESI) available. See DOI: 10.1039/c6tx00101g


**DOI:** 10.1039/c6tx00101g

**Published:** 2016-04-27

**Authors:** Harriet Gaskell, Parveen Sharma, Helen E. Colley, Craig Murdoch, Dominic P. Williams, Steven D. Webb

**Affiliations:** a MRC Centre for Drug Safety Science , Department of Molecular and Clinical Pharmacology , Sherrington Building , Ashton Street and University of Liverpool , L69 3GE , UK . Email: parveen.sharma@liverpool.ac.uk; b AstraZeneca , 310 , Cambridge Science Park , Milton Road , Cambridge , Cambridgeshire , CB4 0FZ , UK; c Academic Unit of Oral and Maxillofacial Pathology , School of Clinical Dentistry , Claremont Crescent and University of Sheffield , Sheffield , S10 2TA , UK; d Department of Mathematical Sciences , Liverpool John Moores University , James Parsons Building , Byrom Street , Liverpool , L3 3AF , UK

## Abstract

More predictive *in vitro* liver models are a critical requirement for preclinical screening of compounds demonstrating hepatotoxic liability. 3D liver spheroids have been shown to have an enhanced functional lifespan compared to 2D monocultures; however a detailed characterisation of spatiotemporal function and structure of spheroids still needs further attention before widespread use in industry. We have developed and characterized the structure and function of a 3D liver spheroid model formed from C3A hepatoma cells. Spheroids were viable and maintained a compact *in vivo*-like structure with zonation features for up to 32 days. MRP2 and Pgp transporters had polarised expression on the canalicular membrane of cells in the spheroids and were able to functionally transport CMFDA substrate into these canalicular structures. Spheroids expressed CYP2E1 and were able to synthesise and secrete albumin and urea to a higher degree than monolayer C3A cultures. Penetration of doxorubicin throughout the spheroid core was demonstrated. Spheroids showed increased susceptibility to hepatotoxins when compared to 2D cultures, with acetaminophen having an IC_50_ of 7.2 mM in spheroids compared to 33.8 mM in monolayer culture. To conclude, we developed an alternative method for creating C3A liver spheroids and demonstrated cellular polarisation and zonation, as well as superior liver-specific functionality and more sensitive toxicological response compared to standard 2D liver models, confirming a more *in vivo*-like liver model.

## Abstract 2

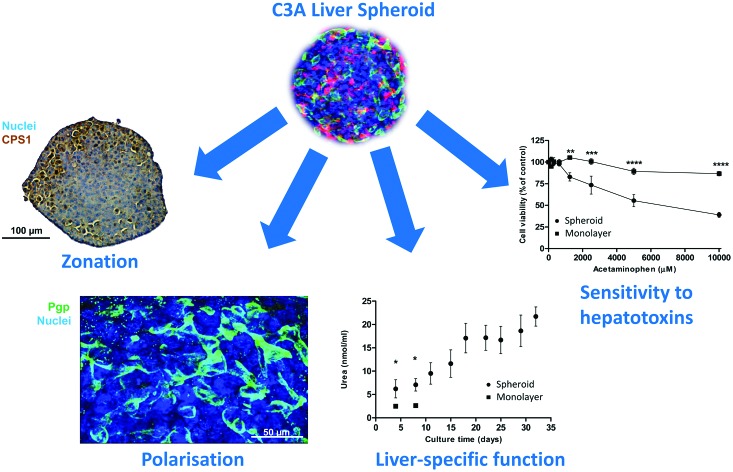
We have developed a method for creating C3A liver spheroids and demonstrated cellular polarisation, zonation as well as increased liver-specific functionality and more predictive toxicological response compared to standard 2D liver models.

## Introduction

Drug-induced liver injury (DILI) is a common side-effect of many therapeutic compounds and often results in the failure of drugs in discovery and development resulting in therapeutics not reaching the market, or being withdrawn.^1^ Exposure to hepatotoxic compounds can result in liver failure, a life threatening condition, usually requiring a liver transplant.^2^,^3^ Predictive *in vitro* liver models are essential during initial drug screening in order to conduct an accurate risk assessment leading to better candidate selection early in the drug discovery and development process. A commonly used *in vitro* model to detect liver injury are freshly isolated human hepatocytes cultured as a monolayer, suspension or sandwich culture.^4^ However the rapid decline in function and viability of these cells *ex vivo* and inter-donor variability are a major limitation to their use.^5^–^8^ Immortalised hepatocarcinoma cell lines, such as HepG2, C3A, Huh7 and HepaRG, have been used as an alternative to freshly isolated hepatocytes since they have extended lifespans and their clonal nature reduces inter-experimental variability.^6^,^9^ However when cultured as a 2D monolayer these cell lines demonstrate low functionality and an altered phenotype compared to human hepatocytes *in vivo.*^4^,^9^–^11^

A second disadvantage of commonly used *in vitro* models is a lack of 3D structure, which has a significant effect on the hepatocyte morphology, function, phenotype, signalling and toxicological response.^12^–^14^ A simple, high-throughput method of culturing cells in 3D is by creating spheroids. Spheroids, also known as microtissues or organoids, are spherical 3D clusters of cells with direct cell–cell contacts that can be formed using a variety of techniques with or without the use of scaffolds.^4^,^15^–^18^ Additionally, spheroids have the potential to be used for long-term repeat dose studies as well as being amenable to high-throughput assays, an advantage for drug screening.^15^,^17^ Unlike more complicated 3D cell culture techniques, such as perfused cultures, bioreactors, scaffold or chip-based systems, spheroids are simple to generate, inexpensive to culture, reproducible and easy to analyse.^4^ The use of spheroids as a model for screening of hepatotoxins is fairly novel; however in other areas of research spheroids are a well-established 3D cell culture technique. So far research into liver spheroids has shown some promising results. When cultured in spheroids isolated human hepatocytes survive for up to 4 weeks, sustaining important functions such as phase I and II enzyme expression, albumin and urea synthesis and expression of liver-specific markers.^19^,^20^ Furthermore bile canaliculi structures can be visualised in the spheroids, indicating that the hepatocytes are polarised and retain their *in vivo*-like morphology and phenotype.^11^,^20^ Additionally, when cultured in spheroids, hepatocytes show cuboidal morphology, enhanced cell–cell contacts and production of extracellular matrix components.^15^,^21^

Multiple studies have also shown that hepatocarcinoma cell lines cultured in 3D have superior liver-specific functionality when compared to monolayer cultures, overcoming one of the main disadvantages of these cells. HepaRG cells have been cultured as spheroids and show a significant improvement in albumin and AboB production, increases in liver specific gene expression and activity, and induction of CYP enzymes when compared to 2D cultured cells.^22^–^24^ Spheroids created from Huh7 cells also display superior function, with expression of phase I and II enzymes as well as polarisation of multiple receptors.^25^ Novel cell lines such as upcyte hepatocytes have also been considered as an alternative to primary cells and express hepatocyte specific markers and functional phase I and II metabolism when cultured as spheroids.^26^ HepG2 cell spheroids have successfully been cultured by multiple research groups and have shown to be viable and functional for at least 28 days, determined by increased albumin production, increased expression of liver specific enzymes and activity of phase I and II enzymes, increased sensitivity to hepatotoxins, bile canaliculi formation and transporter function.^27^–^29^ In addition to this, spheroids formed from HepG2 cells show over-expression of genes involved in xenobiotic and lipid metabolism.^20^,^22^,^23^,^25^,^28^,^30^ This increase in liver-specific function has been shown to raise the sensitivity of HepG2 cells to hepatotoxic compounds and the enhanced lifespan allows for more realistic repeat dosing strategies.^28^ C3A cells are a subclone of HepG2 cells with some advantages, selected for their strong contact-inhibited growth characteristics, as well as high-albumin production, alpha fetoprotein and transferrin synthesis and the ability to grow in glucose deficient media.^31^ This cell line therefore has advantages over others when cultured in spheroids, with a reduced proliferation rate, more representative of the *in vivo* situation.^32^,^33^ Previous studies of C3A spheroids have revealed superior function over 2D cultures,^32^–^34^ however further investigation into the canalicular structures, zonation, liver-specific functionality and toxicological response of this model is necessary before widespread use during drug screening.

3D liver spheroids show promise through enhanced functionality compared to 2D cultures, and could provide a valuable tool for investigating hepatotoxic drugs in pre-clinical safety testing. However, despite the increasing interest and use of 3D liver models, limited research has gone into validating key structural and functional parameters such as cellular morphology and polarisation, secondary structures, oxygen and nutrient diffusion, zonation, drug penetration, liver-specific functionality and the ability to predict hepatotoxic potential.^35^–^37^ In this study we developed and optimised a liver spheroid model using C3A hepatocarcinoma cells and characterised their spatiotemporal structure and function providing essential optimising parameters and an invaluable tool for investigating acute and chronic liver injury.

## Experimental

### Spheroid formation and growth

C3A cells were maintained in EMEM (LGC Standards) supplemented with 10% FBS and 1% penicillin–streptomycin under standard cell culture conditions. For 2D monolayer experiments C3A cells were seeded and left to adhere for 24 hours and confirmed to be 100% confluent before analyses.

Spheroids were created using the liquid overlay technique as previously described.^38^ Briefly, 100 μl of sterile 1.5% Agarose (high gelling temperature-Sigma Aldrich) in EMEM was added per well to flat-bottomed 96-well cell culture plates to form a low-adherence surface. Additionally Ultra Low Adherence plates (ULA-Corning) were used as a comparison. C3A cells were seeded at 500, 750, 1000, 1500, 2000 or 2500 cells per well in 100 μl media and left for 72 hours to form spheroids. Media was renewed twice weekly and spheroids were cultured for up to 32 days. Images of spheroids in culture were taken by phase-contrast microscopy through 4× objective and maximum spheroid diameter measured.

### Histological analysis

Spheroids were washed in PBS, fixed for 1 hour in 4% PFA and embedded in 2% Agarose (low EEO-Sigma Aldrich) in 4% PFA then paraffin embedded. Tissue sections were cut and stained with haematoxylin and eosin (H&E) or as previously described.^39^ Immunohistochemistry for Ki-67, carbamoyl-phosphate synthase 1 (CPS1) and CYP2E1 was carried out by the Department of Veterinary Pathology, Leahurst Campus, University of Liverpool, UK.

### Immunofluorescence analysis of spheroids

Spheroids were transferred to ULA plates, washed three times in PBS and fixed with 4% PFA for 1 hour at 4 °C. Spheroids were washed again then permeabilized with 0.5% Triton X-100 in Tris-Buffered Saline with 0.05% Tween20 (TBST) overnight at 4 °C and then blocked with 0.1% Triton X-100/3% BSA in TBST for 2 hours at room temperature (RT). Primary antibodies Multidrug resistance protein-2 (MRP2-Abcam) and P-glycoprotein (Pgp-Abcam) were diluted 1 : 20 in 0.1% Triton X-100/1% BSA in TBST were incubated with the spheroids overnight at 4 °C. Spheroids underwent three 1 hour washes with 1% Triton X-100 in TBST then incubated with secondary Alexa Fluor 488 Donkey Anti-Mouse antibody (Life Technologies) diluted 1 : 1000, Hoechst diluted 1 : 5000 and Phalloidin 568 diluted 1 : 250 in 0.1% Triton X-100/1% BSA in TBST overnight at 4 °C. Spheroids were finally washed for 1 hour then mounted with Prolong Gold (Life Technologies) onto a glass microscope slide. Maximum intensity projection images of spheroids were taken using a Zeiss Axio Observer microscope with Apoptome using 40× oil objective.

### Immunofluorescence analysis of 2D monolayers

Cells were washed in PBS for 30 min at 4 °C then fixed with 2% PFA for 30 min at 4 °C. Cells were permeabilized with two 15 min washes in 0.2% Tween-20/0.5% Triton X-100 in PBS at 4 °C and blocked for 30 min in 5% BSA/0.2% Tween-20/0.5% Triton X-100 in PBS at room temperature. Primary antibodies MRP2 and Pgp were diluted in 5% BSA/0.2% Tween-20/0.5% Triton X-100 in PBS and incubated with cells overnight at 4 °C. Cells underwent three 15 min washes in 0.2% Tween-20/0.5% Triton X-100 in PBS then incubated with secondary Alexa Fluor 488 Donkey Anti-Mouse antibody diluted 1 : 1000, Hoechst diluted 1 : 5000 and Phalloidin diluted 1 : 250 in 5% BSA/0.2% Tween-20/0.5% Triton X-100 in PBS for 1 hour at room temperature. Cells underwent three 15 min washes in PBS then were mounted with Prolong gold onto a glass microscope slide. Images were taken using a Zeiss Axio Observer microscope with Apoptome using 40× oil objective.

### Analysis of transporter function

Spheroids and monolayers were incubated with 5 μM 5-chloromethylfluorescein diacetate (CMFDA-Life Technologies) with or without 25 μM MK571 (MRP inhibitor) and 12.5 μM PSC833 (Pgp inhibitor) in EMEM for 30 min at 37 °C. CMFDA is membrane permeable until it enters cell and is converted to glutathione-methylfluorescein (GSMF), a cell impermeable substrate for MRP and Pgp.^40^ Cells and spheroids were washed in PBS and prepared for immunofluorescence as described above.

### Quantification of albumin and urea production

Albumin and urea in spheroid and monolayer supernatant were quantified using Albumin Human ELISA Kit (Abcam) and Urea Assay kit (Abcam) respectively, according to the manufacturer's protocol. Samples were collected 4 days after media change, twice weekly over 32 days. Data was normalised to account for differences in cell number.

### Quantification of Keratin 18

Total Keratin 18 and cleaved Keratin 18 in spheroid supernatant were quantified using M65 and M30 ELISA kits (Peviva) respectively, according to the manufacturer's protocol. Apoptotic cell death was represented by the Cleaved Keratin 18 concentration, total cell death represented by total Keratin 18, and necrotic cell death calculated from the total cell death minus apoptotic cell death.

### Visualisation of compound penetration

Spheroids were treated with 3 μg per ml doxorubicin for 24 hours then washed in PBS, fixed in 4% PFA for 1 hour then incubated with Hoechst diluted 1 : 5000 in 0.1% Triton X-100 /1% BSA in TBST for 1 hour. Images were taken using a Zeiss LightSheet Z.1 microscope.

### Toxicological analysis

Spheroids were treated at day 3 of culture with hepatotoxic compounds acetaminophen, fialuridine, diclofenac and trovafloxacin diluted in 0.5% DMSO in EMEM for 4 days with repeat dosing on day 2. Monolayer C3A cells were treated with compounds for 24 hours. Cell viability was analysed using Cell Titer-Glo assay (Promega) according to the manufacturer's instructions and plotted as a percentage of untreated control.

### Statistical analysis

Data expressed are representative of at least three independent experiments (*n* = 3) in triplicate and represented as mean ± standard error. Graphs and statistical analysis were performed using GraphPad Prism 5 (Graphpad software, San Diego, CA, USA).

## Results

### Optimisation of spheroid starting cell number

**Fig. 1:**
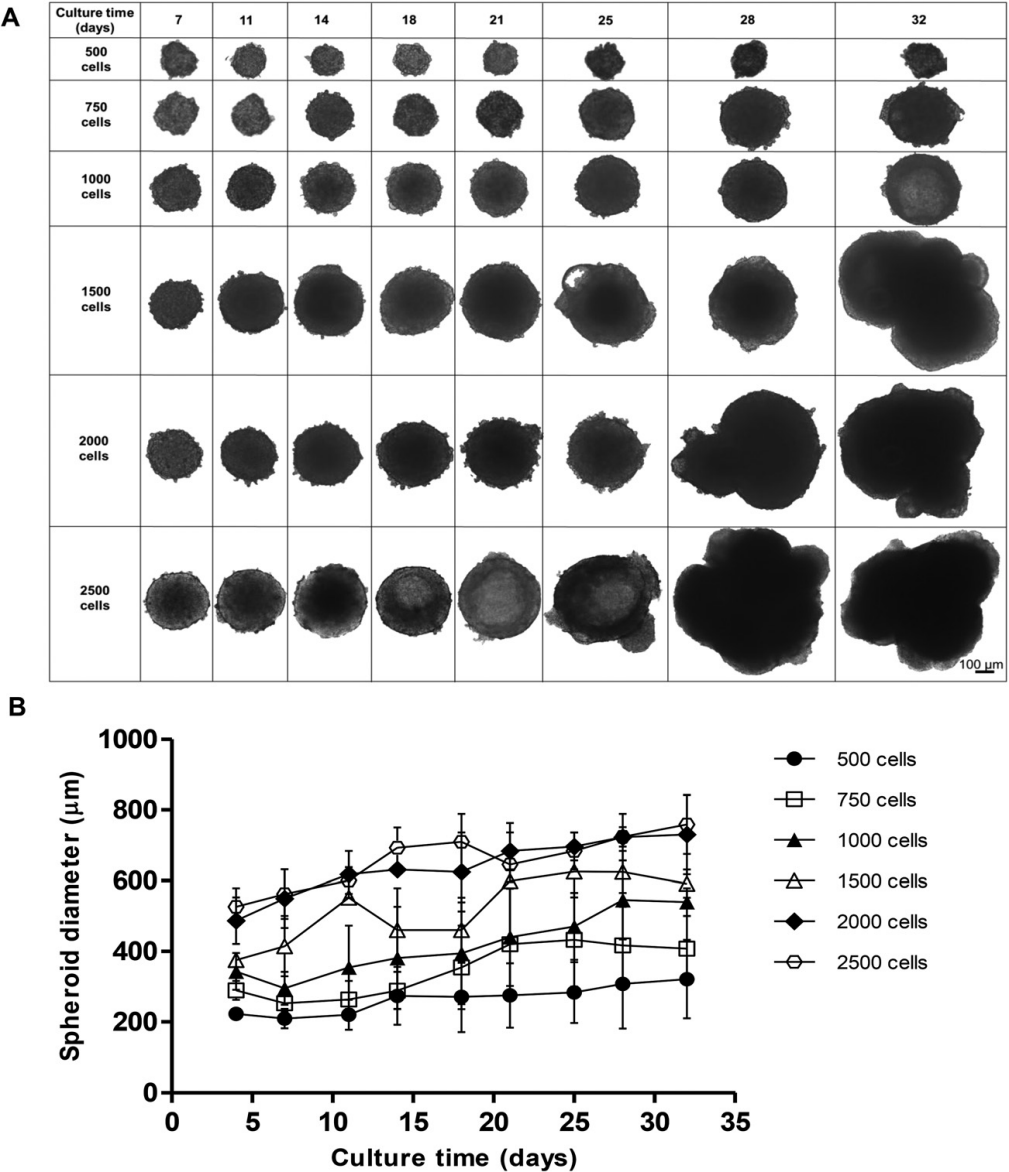
Effect of starting cell number on spheroid size and morphology. Spheroids were created from 500, 750, 1000, 1500, 2000 and 2500 C3A cells and cultured for 32 days. (A) Phase-contrast images of spheroids, images taken at day 7, 11, 14, 18, 21, 25, 28, 32. Scale bar = 100 μm. (B) Growth curve of spheroids over 32 days. Spheroid diameter (μm) was plotted against culture time (days). Data are represented as mean ± standard error (*n* = 3 in triplicate).

We initially compared two different scaffold-free approaches to create our C3A spheroids, the liquid-overlay technique and ULA plates. Despite having similar growth characteristics, spheroids created on ULA plates had an irregular, non-spherical structure with a less defined outer perimeter compared to those formed using the liquid overlay technique, which were consistently uniform and spherical (ESI Fig. 1†). We therefore chose to perform all future experiments using the liquid-overlay technique. We then assessed how different starting cell numbers may affect spheroid formation, size and shape over time. Spheroids were formed from a starting cell number of 500, 750, 1000, 1500, 2000 and 2500 C3A cells and cultured for 32 days. Brightfield microscopy was used to measure spheroid diameter and morphology twice weekly (Fig. 1).

All starting cell numbers resulted in the formation of spheroids of varying sizes, with all of the cells in each well aggregating to form a single spheroid. Spheroids created from 500 cells gradually increased in diameter over 32 days from 237.0 ± 14.3 nm to 432.9 ± 111.4 nm in diameter, however these spheroids were less uniform and less stable in shape, resulting in disaggregation of some cells (Fig. 1A and B). However, spheroids with a starting cell number of 750 or 1000 cells steadily grew over 32 days, 289.4 ± 26.5 nm and 343.2 ± 73.3 nm at day 4 and increasing to 407.7 ± 92.3 nm and 539.5 ± 39.5 nm at day 32 respectively, and maintained a uniform spherical shape over the course of the culture. Spheroids created with higher starting cell numbers, 1500, 2000 or 2500 cells, increased in diameter more rapidly, reaching much larger diameters of 624.5 ± 59.5 nm, 730.0 ± 112.0 nm and 759.1 ± 83.5 nm, as well as becoming irregular in shape (Fig. 1A and B). From this data, 750 cells was determined to be an optimal cell number for creating spheroids, as these spheroids stayed the most uniform in shape over 32 days, with little variation in size and the smallest diameter. 2500 cell spheroids have been subsequently used in our analysis as a comparison.

### Internal spheroid structure

**Fig. 2:**
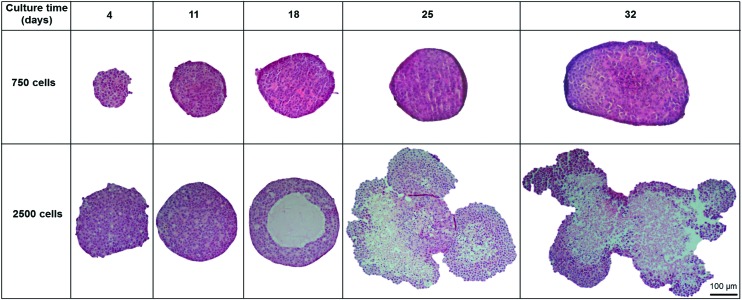
Spheroid internal structure and morphology. Spheroids were created on liquid-overlay plates from 750 or 2500 C3A cells and fixed at day 4, 11, 18, 25 and 32 of culture, paraffin embedded, sectioned, stained with H&E. Images represent mid-sections through the spheroids. Scale bar = 100 μm.

In order to analyse the internal structure, H&E staining was performed on spheroids with 750 and 2500 cell starting numbers to visualise internal cell morphology and arrangement over 32 days (Fig. 2). Staining of sectioned spheroids with a starting cell number 750 cells revealed a compact, uniform structure throughout the spheroid, with a defined outer perimeter (Fig. 2). Cells within the spheroids had a cuboidal 3D morphology with direct cell–cell contacts, similar to that seen in a human liver. Correlating with cell growth data, the 750 cell spheroids were seen to gradually increase in size, yet stayed uniformly spherical with limited degrees of necrosis up to day 32. However in the larger 2500 cell spheroids small patches of cell death started to occur around day 14 and, by day 18, a necrotic core had formed. This was confirmed by analysis of cell death biomarker release, revealing significantly higher levels of necrosis in larger spheroids (ESI Fig. 2†). Additionally, by day 25 the 2500 cell spheroids became misshapen and their growth started to rapidly increase, resulting in the spheroids disaggregating and losing structural integrity (Fig. 2).

**Fig. 3:**
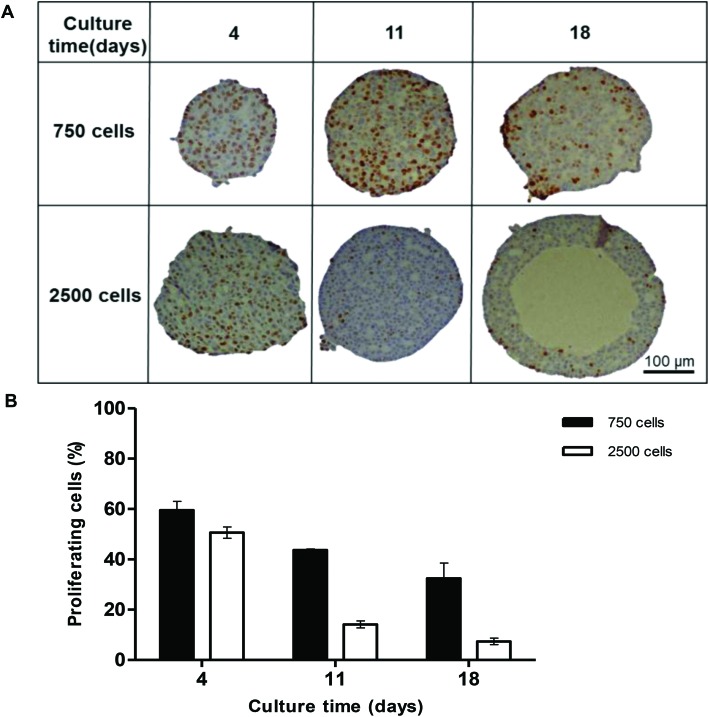
Proliferation of cells within the spheroids. Spheroids were created on liquid-overlay plates from 750 or 2500 C3A cells and fixed at day 4, 11 and 18 of culture, paraffin embedded, sectioned and stained with ki67 (brown) to stain proliferating cells, and haematoxylin (blue) to stain the nuclei. (A) Images of spheroid mid-sections. Scale bar = 100 μm; (B) Graph showing proliferating cells (%) plotted against culture time (days). Data are represented as mean ± standard error (*n* = 3).

Proliferation of the cells inside the spheroid was analysed over the first 18 days of culture using Ki-67 staining in spheroids with 750 and 2500 starting cell numbers (Fig. 3A). Nuclei stain blue with haematoxylin and proliferating cells appear brown, stained with Ki-67. At day 4, 59.5 ± 3.5% and 50.6 ± 2.3% of the cells in the spheroids were proliferating for 750 and 2500 starting cell numbers respectively (Fig. 3A and B). After 18 days in culture, fewer cells were seen to be proliferating, 32.4 ± 6.1% and 7.4 ± 1.3% respectively, with proliferating cells mainly located nearer to the periphery of the spheroid (Fig. 3A).

**Fig. 4:**
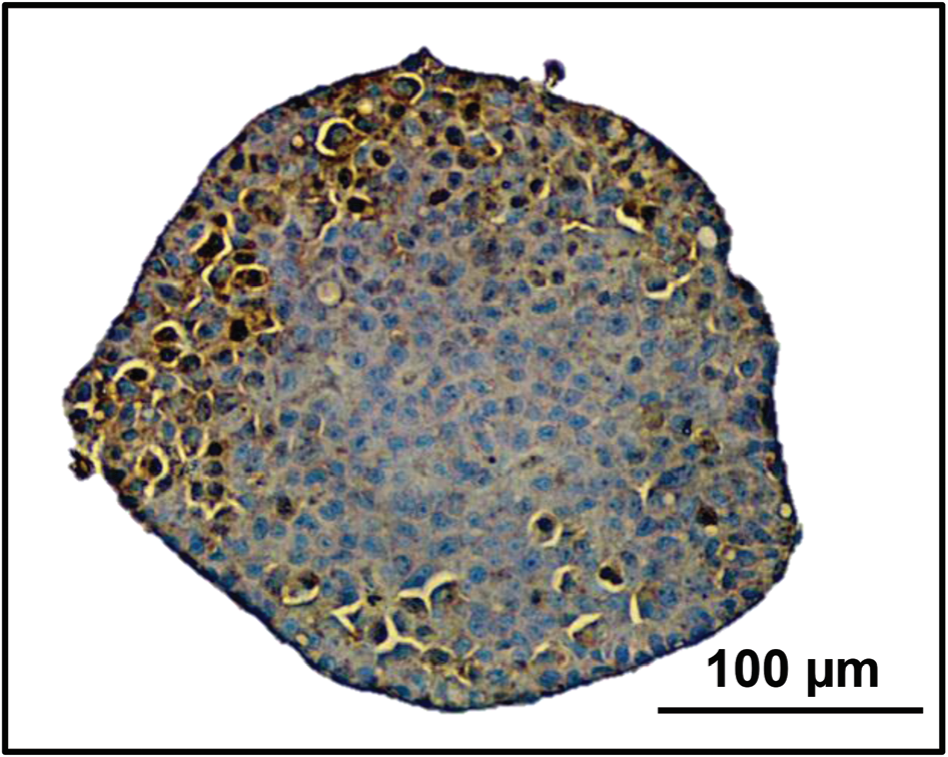
Zonation in spheroids. Spheroids were created on liquid-overlay plates from 750 cells and fixed at day 18 of culture, paraffin embedded, sectioned and stained with CPS1, a periportal marker (brown), and haematoxylin (blue) to stain the nuclei. Scale bar = 100 μm.

The liver lobule can be classed as three regions, periportal, transitional and perivenous regions which display zonation due to the gradient of oxygen and nutrients available from the sinusoidal microvessels.^41^ CPS1 can be utilised as a zonation marker for periportal areas of the liver.^42^ We stained spheroids cultured for 18 days for CPS1 and found that it was expressed near the periphery of the spheroid, an area with the highest oxygen concentrations, similar to the periportal area of the liver lobule (Fig. 4).

### Cellular polarisation in spheroids

**Fig. 5:**
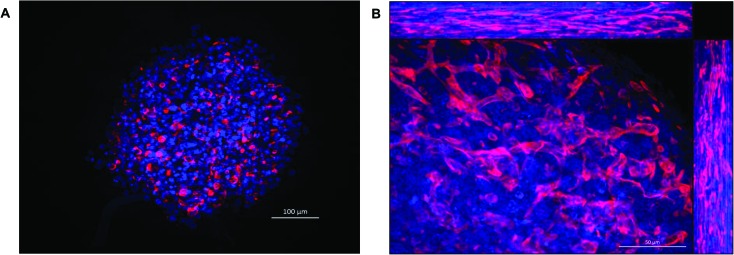
Secondary structure formation in spheroids. Spheroids were cultured on liquid-overlay plates for (A) 4 days or; (B) 18 days, fixed and stained with Hoechst (blue) to stain the nuclei and phalloidin (red) to stain F-actin. Maximum intensity projection images were taken using a Zeiss Axio Observer microscope. Scale bars = 100 μm and 50 μm respectively.

One of the key features of hepatocytes is their ability to polarise. This involves the formation of bile canaliculi between adjacent cells, as well as the correct localisation of key transporters to either the apical or canalicular membranes.^4^,^43^ We used immunofluorescence to analyse the internal structure of the spheroids over time. Spheroids were fixed and stained with phalloidin (red) to visualise F-actin structures, by confocal microscopy (Fig. 5). After 4 days of culture, F-actin filaments were observed forming between cells within the spheroid (Fig. 5A). After 11 days in culture, these larger structures could be seen joining together to create a network throughout the spheroid (Fig. 5B).

**Fig. 6:**
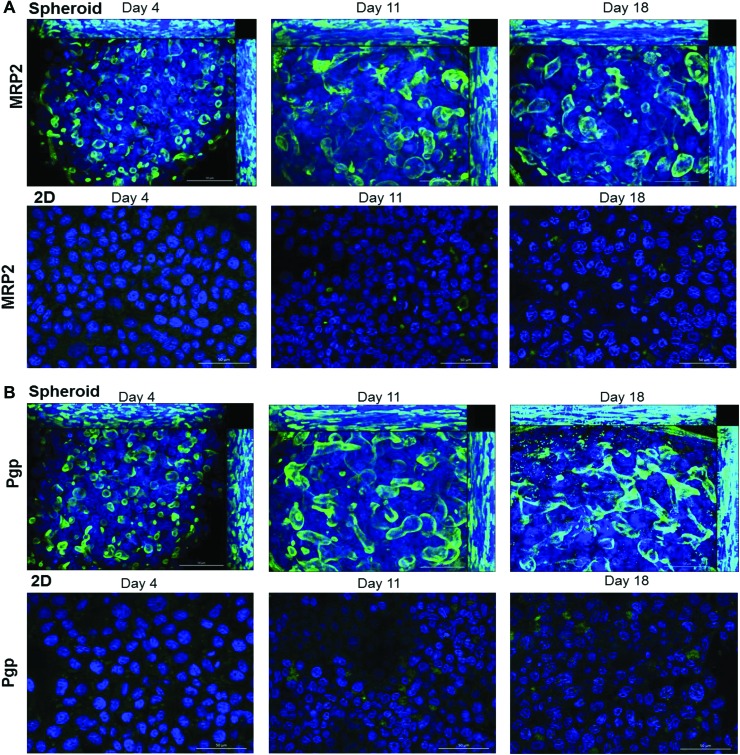
Transporter polarisation in spheroids. Spheroids were created from 750 C3A cells by liquid-overlay technique and compared to C3A cells cultured in a 2D monolayer. Samples were fixed at day 4, 11 and 18 of culture. Immunofluorescent staining was performed for the canalicular transporter (A) MRP2 (green) or; (B) Pgp (green) and Hoechst (blue) to stain the nuclei. Maximum intensity projection images were taken using a Zeiss Axio Observer microscope. Scale bars = 50 μm.

MRP2 and Pgp are transporters localised to the canalicular membrane of hepatocytes and transport organic anions and efflux lipophilic cations from the cell respectively.^44^ The expression of these transporters were used to confirm whether the actin structures observed were the result of cellular polarisation and the formation of bile canaliculi. Spheroids and 2D cultured C3A cells were analysed over 18 days for MRP2 (Fig. 6A) and Pgp (Fig. 6B) expression. The staining pattern of these transporters emulated that seen with the phalloidin (Fig. 5). The secondary structures could be seen forming from day 4 of culture until at least day 18, regardless of spheroid size (ESI Fig. 3†), and appeared to elongate and interconnect over time, forming an interconnected network of canalicular structures (Fig. 6). The same staining pattern for MRP2 and Pgp was not seen in a 2D monolayer culture (Fig. 6).

### Confirmation of liver-like function in spheroids

**Fig. 7:**
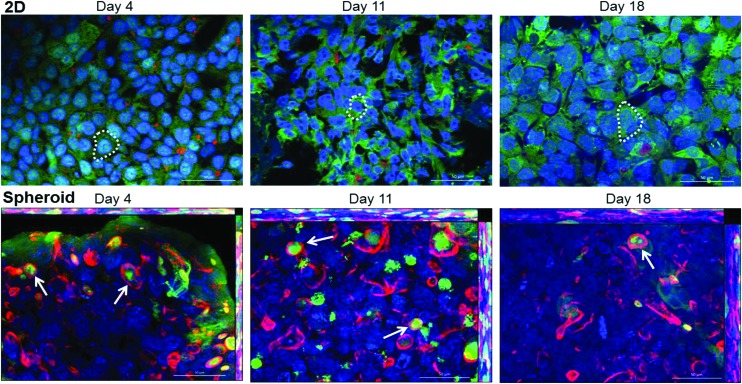
Transporter function in spheroids. Spheroids were created from 750 C3A cells on liquid-overlay plates or C3A cells were cultured in a 20 monolayer on glass coverslips for 4, 11 or 18 days and then incubated with CMFDA (green) an MRP and Pgp transporter substrate for 30 min, washed, fixed and stained with Hoechst (blue) to stain the nuclei and phalloidin (red) to stain F-actin. Images were taken using a Zeiss Axio Observer microscope. Dotted line indicates an example of a cell where CMDFA is retained within the cell cytoplasm. Arrow indicates the canalicular-like structures containing CMFDA. Scale bars = 50 μm.

Transporter functionality was determined using fluorescently labelled CMFDA. This compound can passively enter cells but can only be effluxed from cells *via* active transport through MRP2 or Pgp.^40^ 2D monolayer C3A cells retained CMFDA (green) within the cell cytoplasm (Fig. 7). However in the spheroids there was limited retention of CMFDA within cell cytoplasm and an accumulation and co-localisation of the compound within secondary canalicular-like structures, shown by F-actin in red (Fig. 7). Blocking MRP and Pgp transporters resulted in CMFDA being retained in the cell cytoplasm (ESI Fig. 4†). This suggests that in the spheroids CMFDA was actively transported out of the cells by MRP2 and Pgp into the secondary structures, providing evidence that these transporters are functioning correctly.

**Fig. 8:**
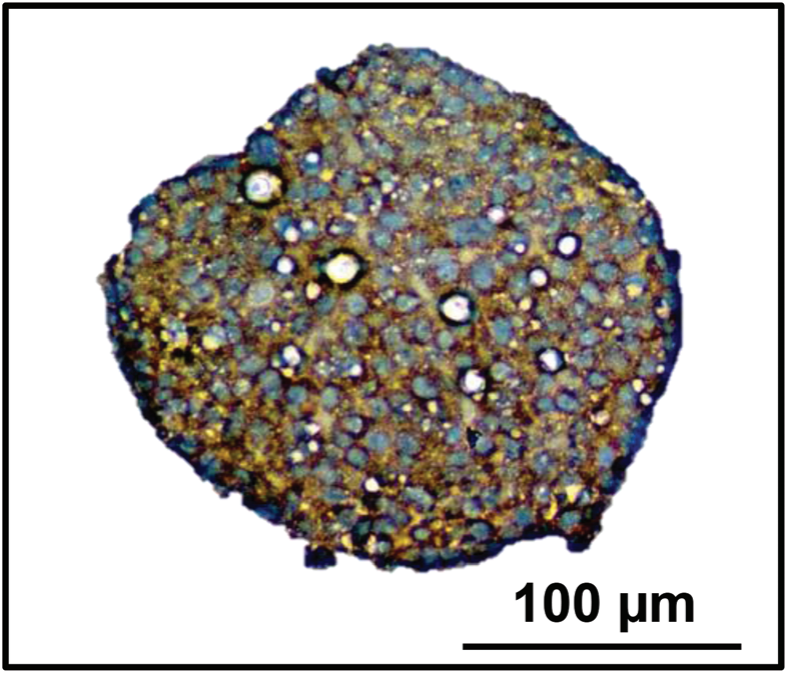
CYP2E1 enzyme expression in spheroids. Spheroids were created on liquid-overlay plates from 750 cells and fixed at day 11 of culture, paraffin embedded, sectioned and stained with CYP2E1 (brown) and haematoxylin (blue) to stain the nuclei. Scale bar = 100 μm.

In order to determine the presence of key liver-specific enzymes we stained for CYP2E1, an important phase I metabolic enzyme in the human liver. CYP2E1 was clearly expressed throughout the spheroid, indicating that the spheroids possess this liver-specific functional marker (Fig. 8).

**Fig. 9:**
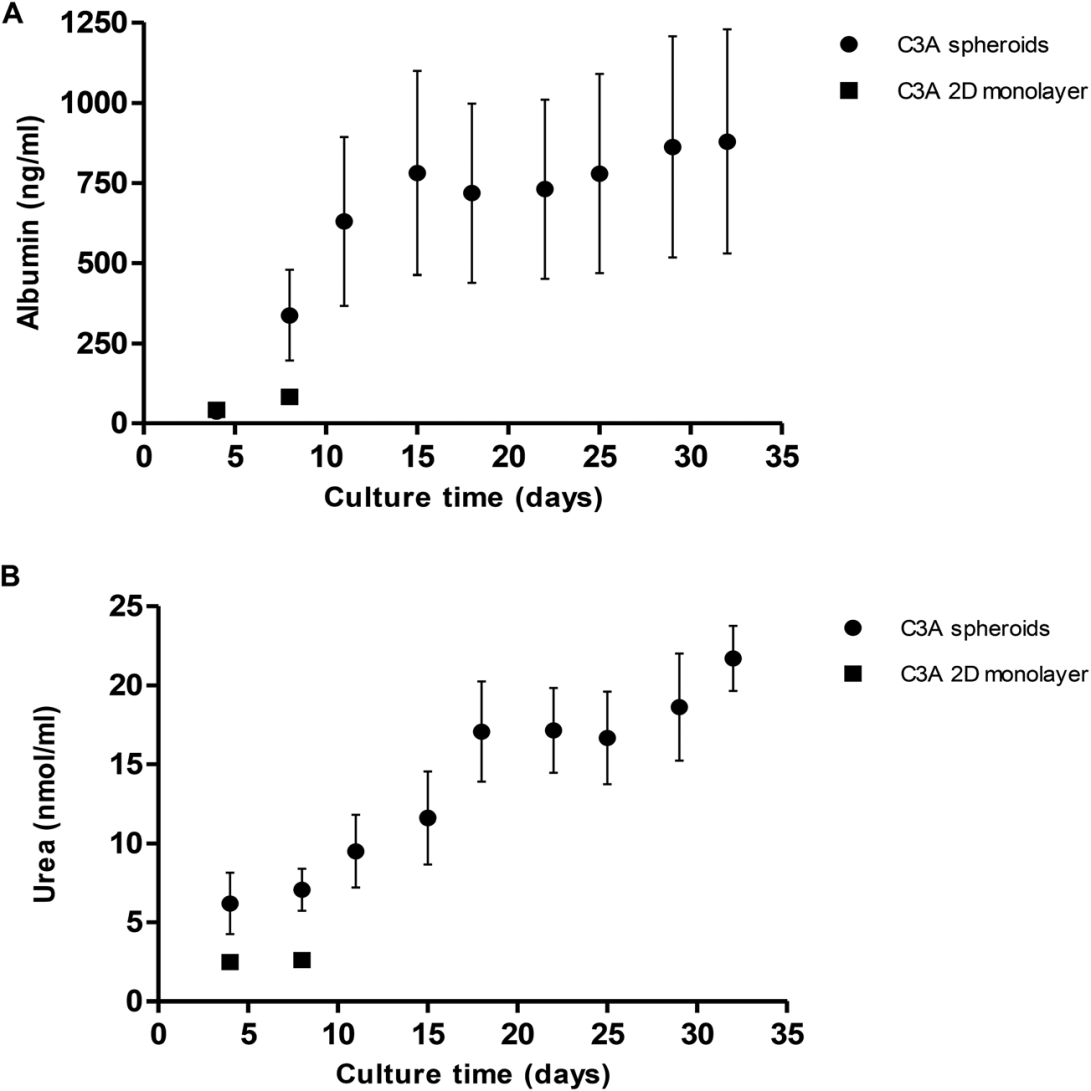
Albumin and urea production in spheroids. Spheroids were created from 1000 C3A cells on liquid-overlay plates and C3A monolayer cultures used as a comparison. (A) Albumin and, (B) urea release were quantified in spheroid (black circle) and monolayer (black square) supernatant and plotted again culture time (days). Data are represented as mean ± standard error (*n* = 3 in triplicate).

Albumin and urea production from spheroids was quantified over 32 days. Albumin production gradually increased from 36.9 ± 15.5 ng ml^–1^ at day 4 of culture to 878.9 ± 349.6 ng ml^–1^ at day 32 (Fig. 9A). We compared this to 2D monolayer cultured C3A cells which were seen to produce a maximum 82.3 ± 5.4 ng ml^–1^ albumin, significantly less than the values obtained in 3D cultures. Similarly, urea production steadily increased over 32 days from 6.2 ± 1.9 nmol ml^–1^ to 21.7 ± 2.0 nmol ml^–1^ (Fig. 9B). Monolayer cultured C3A cells produced significantly less urea, with a maximum of 2.6 ± 0.2 nmol ml^–1^.

### Drug penetration throughout the spheroid

**Fig. 10:**
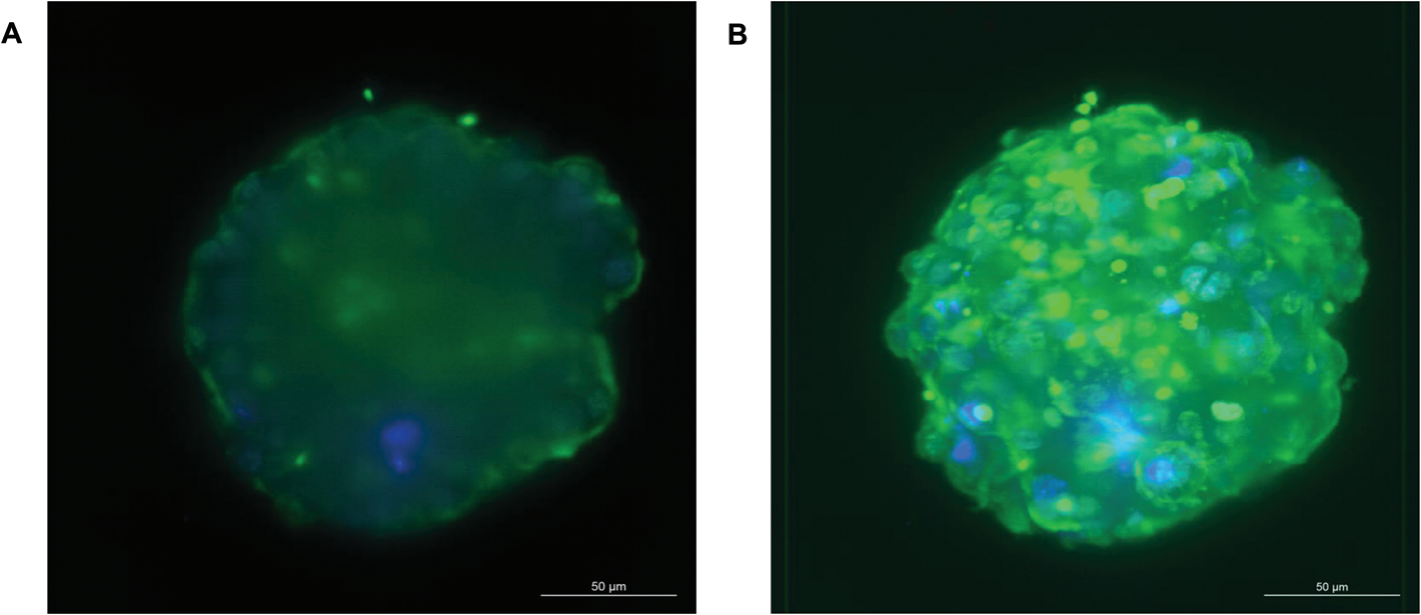
Drug penetration throughout the spheroid. Spheroids were cultured on liquid-overlay plates for 11 days, treated with 3 μg per ml doxorubicin (green) for 24 hours, fixed, stained with Hoechst (blue) to stain the nuclei and imaged by Zeiss LightSheet Z.1 microscope. (A) Represents a mid-section through the spheroid and; (B) a maximum projection image. Scale bars = 50 μm.

The assessment of acute and chronic drug toxicity is one of the key applications of 3D spheroids. We next wanted to confirm compound penetration and ascertain whether or not all the cells within the spheroids are being exposed to compounds added exogenously. In order to analyse compound penetration 11 day old 750 cell spheroids (estimated to be around 263 μm in diameter, Fig. 1B) were treated with doxorubicin, an autofluorescent chemotherapeutic compound with a molecular mass of 543 g mol^–1^ and a log *P* of 1.27, and imaged using LightSheet microscopy. Separate images were taken throughout the *z*-plane of the spheroid to visualise the entire spheroid core. Fig. 10 shows the spheroid after treatment with doxorubicin (green) for 24 hours, with nuclei shown in blue. Doxorubicin can be seen fluorescing throughout the spheroid core in the median section through the spheroid (Fig. 10A), as well as throughout the periphery (Fig. 10B), thus confirming that this compound is in contact with every cell in the spheroid. From this we can assume that the majority of cells in the spheroid will be exposed to similar concentrations of an exogenously treated compound.

### Toxicological analysis

**Fig. 11:**
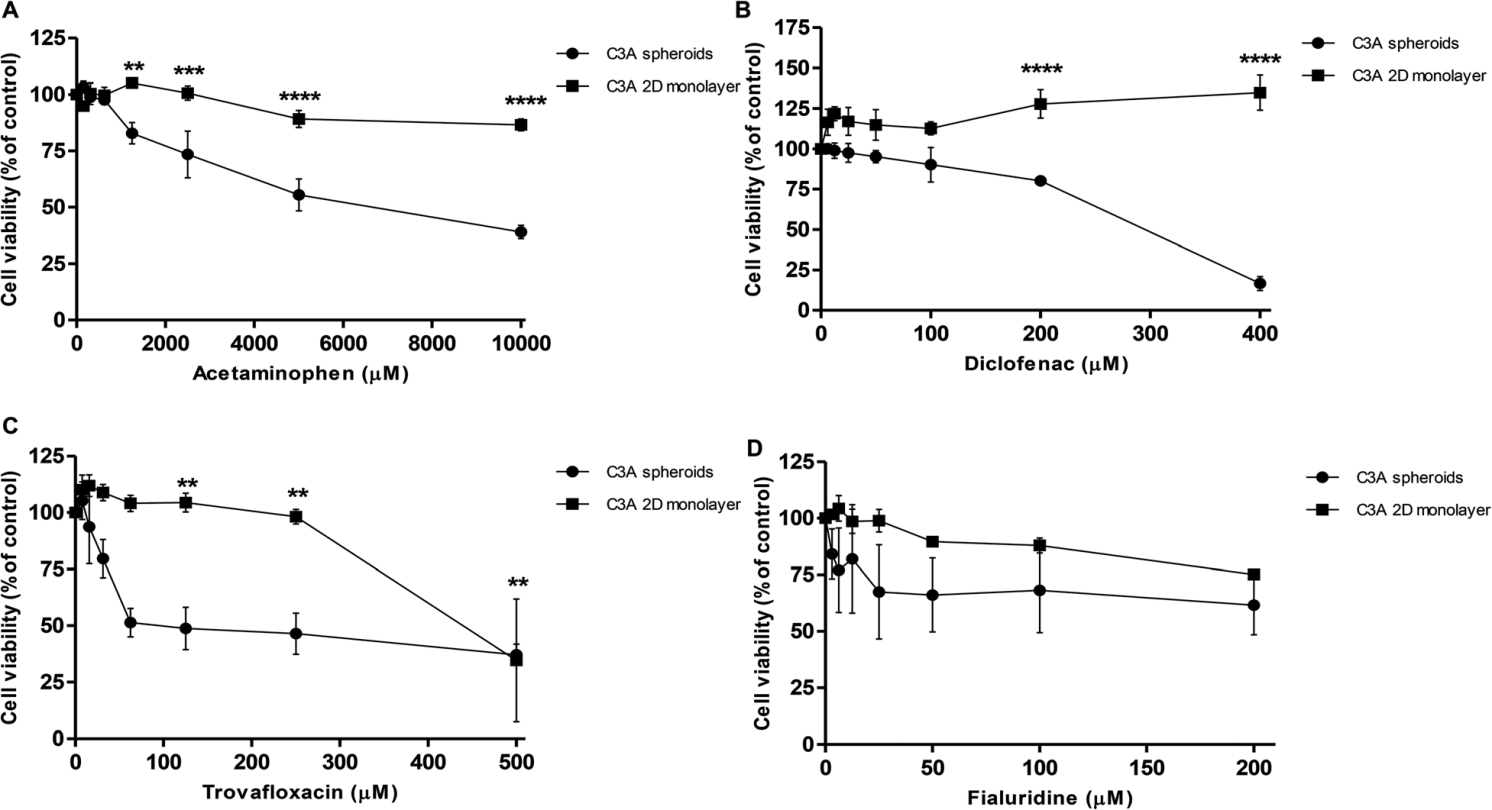
Spheroids show sensitivity to hepatotoxins. Spheroids were created from 1000 C3A cells by liquid-overlay technique and cultured for 3 days, using 2D monolayer C3A cells as a comparison, and treated with (A) acetaminophen; (B) diclofenac; (C) trovafloxacin and (D) fialuridine. Cell viability was analysed and plotted as a percentage of untreated control. Data are represented as mean ± standard error. ****p < 0.0001, ***p < 0.001, **p < 0.01, *<0.05 (*n* = 3 in triplicate).

Spheroids were treated with varying concentrations of hepatotoxic compounds and compared to C3A cells cultured as a 2D monolayer. A repeat-dosing regimen could be used for spheroids, as we have previously confirmed their viability and functionality over this period; however 2D monolayer cultures were given acute doses as these cells become over-confluent and loose viability once cultured for over 48 hours. Dose-dependent reduction in cell viability was observed in the spheroids in response to all 4 hepatotoxins (Fig. 11). Table 1 lists the IC_50_ values calculated for each compound in C3A spheroids compared to monolayer cultures. For example acetaminophen was significantly more toxic to spheroids than monolayer cultures, with an IC_50_ value of 7.2 mM in spheroids and 33.8 mM in monolayers. Spheroids were also significantly more sensitive to trovafloxacin, with an IC_50_ value of 65 μM in spheroids compared to 440 μM in 2D cultures, as well as fialuridine, which had an IC_50_ value of 206 μM in 3D culture and 380 μM in 2D (Table 1). In the monolayer cultures diclofenac did not have a toxic response at the concentrations used, although the IC_50_ value is reported to be around 763 μM in HepG2 monolayer cultures^45^ and was found to be 295 μM in spheroid cultures and significantly more toxic (Fig. 11).

**Table 1:** Toxicological analysis of spheroids. Spheroids were created from 1000 C3A cells by liquid-overlay technique, using 2D monolayer C3A cells as a comparison, and treated with 4 hepatotoxins. Cell viability was analysed IC_50_ values calculated. Data are represented as mean values (*n* = 3 in triplicate)

Compound	IC_50_ value in C3A spheroids (μM)	IC_50_ value in C3A 2D monolayer (μM)
Paracetamol	7212	33 826
Diclofenac	295	Non toxic
Trovafloxacin	65	440
Fialuridine	206	380

## Discussion

3D *in vitro* models are becoming more widely used when investigating drug toxicity. Numerous companies now offer spheroids to be shipped ready for use in drug toxicity testing. Research has shown the potential for liver spheroid models to predict hepatotoxicity more precisely than commonly used 2D liver models, as well as spheroids being more amenable to high-throughput screening and long-term repeat dose studies. However many structural and functional characteristics of liver spheroids have not been fully investigated, as well as minimal comparison between the functionality and toxicological predictivity of liver spheroids and other 2D models.

We have developed a technique for creating liver spheroids from the C3A cell line. C3A cells, a derivative of HepG2 cells, were chosen for this model as they exhibit strong contact-inhibited growth characteristics, therefore when cultured in a spheroid these cells do not proliferate to the same extent as other hepatocarcinoma cell lines.^33^ This emulates primary cells which bear limited proliferation capacity. Other advantages include unlimited lifespan, stable phenotype and absence of donor variation.^6^,^9^ As with other liver cell lines, C3A cells have clear disadvantages when cultured in 2D, including limited expression of metabolizing enzymes and lack of liver-specific functions,^9^,^10^ however research is now emerging that indicates 3D culture may overcome some of these key issues. Previous work has been published on C3A spheroids, such as Wrzesinski's group, who used AggreWell plates to create their aggregates and cultured them in micro-gravity bioreactor.^32^–^34^ Extensive characterisation of spheroid growth and viability was performed, as well as determining increases in urea and cholesterol synthesis over 42 days of culture, changes in gene expression and a toxicological response comparable to *in vivo.*^32^–^34^ We adopted an alternative scaffold free approach for creating spheroids in order create direct cell–cell contacts and to reduce any deleterious effects of extracellular matrix components or scaffolds.^12^–^14^

In this study, we created and characterised uniform, reproducible C3A spheroids using the liquid-overlay technique, which has not been previously reported to have been used with this cell line. We found that this technique was superior to the ULA plate's method, a similar scaffold-free technique, creating more uniform, spherical aggregates. The liquid-overlay technique was reliable and reproducible, forming structurally stable spheroids in every experiment in every well. This method was a highly user-friendly system as it does not require an extensive tissue engineering background, and the cost-effectiveness of the model and amenability to both high-throughput and repeat-dose studies makes it a promising model for toxicological studies. Spheroids created from 750 cells were viable for at least 32 days and remained relatively small, with a maximum diameter of 407.8 ± 92.3 nm, and uniformity in shape and size. Proliferation of cells within the spheroids decreased over time, similar to that seen with HepG2 spheroids.^28^ Histological analysis revealed a cuboidal cell morphology within the spheroids, with direct cell–cell contacts similar to an *in vivo* liver structure, unlike in 2D liver cell models where the cells have altered cell morphology, including an elongated and flattened structure, with cell contacts only in one plane, therefore limiting cell signalling.^12^,^14^ Additionally our spheroids recapitulate the zonation of the liver that occurs *in vivo* due to oxygen and nutrient gradients. We confirmed this by staining for the periportal marker CPS1, which is expressed in areas nearest to the oxygenated blood supply in the liver and can be seen staining the peripheral regions on the spheroid in a similar expression pattern, indicating spatiotemporal similarity compared to the human liver.^42^

One disadvantage of using spheroids for drug testing is that cell death can occur within the spheroid core, distorting toxicity data. It has been suggested that oxygen and nutrients can diffuse through tissue approximately 100 μm,^35^–^37^ however little research has gone in to specifically determining the size or time at which liver spheroids develop necrosis in the core, which may well depend of the cell type, cell number, scaffold interactions and culture conditions. We have found in our model that 750 C3A cells is an optimum starting cell number and a culture period of 32 days in which liver spheroids do not possess a visible necrotic core, with minimal apoptosis and necrosis, reaching a size of approximately 400 μm. However we also revealed that once the C3A spheroids developed using our method exceed 700 μm in diameter a necrotic core develops and increasing levels of necrotic biomarkers are observed. Henceforth, C3A spheroids exceeding this critical size cannot be used to accurately determine the effect of a toxicological compound since they are likely to have a pre-existing necrotic core which would give unrepresentative results. The size of the spheroid is therefore an essential parameter that should be taken into account in toxicological studies.

*In vivo*, hepatocytes are structurally and functionally polarised,^4^,^43^ and we have recapitulated this phenotype in our spheroids. Secondary structures composed of actin can be observed forming enhanced networks throughout the spheroids, which has been previously reported in other studies.^28^,^46^ We confirmed that these structures were a result of cellular polarisation by staining for MRP2 and Pgp, transporters known to be expressed and localised to the canalicular membrane of hepatocytes *in vivo.*^43^ MRP2 transporter function has been proven in HepG2 spheroids,^29^ but has not previously been investigated in a C3A spheroid model. MRP2 and Pgp transporter function was confirmed in our C3A spheroids in an experiment utilising CMFDA, a substrate actively transported out of cells by functional canalicular transporters.^40^ We witnessed accumulation of CMFDA in the bile canalicular structures of our spheroids, with little remaining within the cell, revealing that MRP2 and Pgp transporters in the spheroid are functional. This was not observed for monolayer cultured C3A cells, suggesting lack of expression of MRP2 and Pgp, improper polarisation or localisation, or inactive transporters when cultured in 2D. As another indicator of liver-specific function, we stained for CYP2E1,^42^ an important phase I metabolic enzyme. We confirmed expression of CYP2E1 throughout the spheroid at day 18 of culture, similar to HepG2 spheroids where maximal expression was seen on day 21 to 28 of culture.^28^ We additionally found that our spheroids were able to synthesise and secrete albumin and urea, with a gradual increase in production over 32 days of culture and around ten times more than that produced in 2D monolayer C3A cells. Albumin and urea production are important physiological functions of hepatocytes *in vivo*, therefore this data provided further evidence of liver-specific functionality in our spheroids, as and builds on the finding that cells cultured in 3D have superior functionality to those cultured in 2D.^47^

One complication when comparing 2D monocultures to spheroids is whether or not the cells in both models are exposed to the same concentrations of drug compounds. By using LightSheet microscopy we have confirmed that the chemotherapeutic drug doxorubicin can penetrate into cells in the core of a spheroid with a diameter of around 263 μm. This allows us to assume that all cells within the treated spheroids were exposed to similar concentrations of doxorubicin, although the exact concentration cannot be quantified using this technique. We can however predict that other compounds with similar physicochemical properties to doxorubicin (molecular mass 543 g mol^–1^, log *P* of 1.27) would also be able to penetrate the C3A spheroids, enabling us to utilise this model for drug toxicology studies.

We analysed the toxicological predictivity of our C3A spheroids by treating them with a variety of hepatotoxins, each of which cause toxicity by a different mechanism,^48^–^50^ and compared this to standard 2D monolayer cultures of the same cell type. We were able to utilise a repeat-dosing strategy in our spheroids as we had previously confirmed that they were viable and functional over this time course. IC_50_ values were calculated, as this is an industry standard for determining whether or not an *in vitro* model can detect a toxic response. Spheroids were found to be more susceptible to toxicity from these hepatotoxic compounds than 2D cultures, indicated by lower IC_50_ values in the spheroid model. This proves that the 3D spheroid model is more sensitive to hepatotoxins than standard 2D models and superior at predicting whether or not a compound is likely to cause hepatotoxicity in humans.

## Conclusion

Spheroids have promise to be adopted as an *in vitro* liver model for safety screening, displaying an enhanced functional lifespan for repeat-dose studies and amenability to high-throughput screening. We have successfully developed a reproducible technique for creating uniform C3A spheroids and characterised their growth and proliferation characteristics over 32 days. Our spheroids display an *in vivo*-like structure, direct 3D cell–cell contacts, zonation and structural and functional polarisation. We additionally confirmed liver-specific functions such as the ability to synthesise and secrete albumin and urea, functional canalicular transporters and CYP2E1 expression. Toxicological analysis of 4 known hepatotoxins indicated that our spheroid model can predict hepatotoxic potential with a higher sensitivity than standard 2D monolayer cultures.

## Supplementary Material

Supplementary information
