# Supplementary material for: Characterization of a functional C3A liver spheroid model
Source: Toxicol Res (Camb). 2016 Apr 27;5(4):1053–65. doi: 10.1039/c6tx00101g (PMC5047049; doi:10.1039/c6tx00101g)
Supplement: Supplementary file 1 [file TX-005-C6TX00101G-s001.pdf]

Electronic Supplementary Material (ESI) for Toxicology Research.
This journal is © The Royal Society of Chemistry 2016

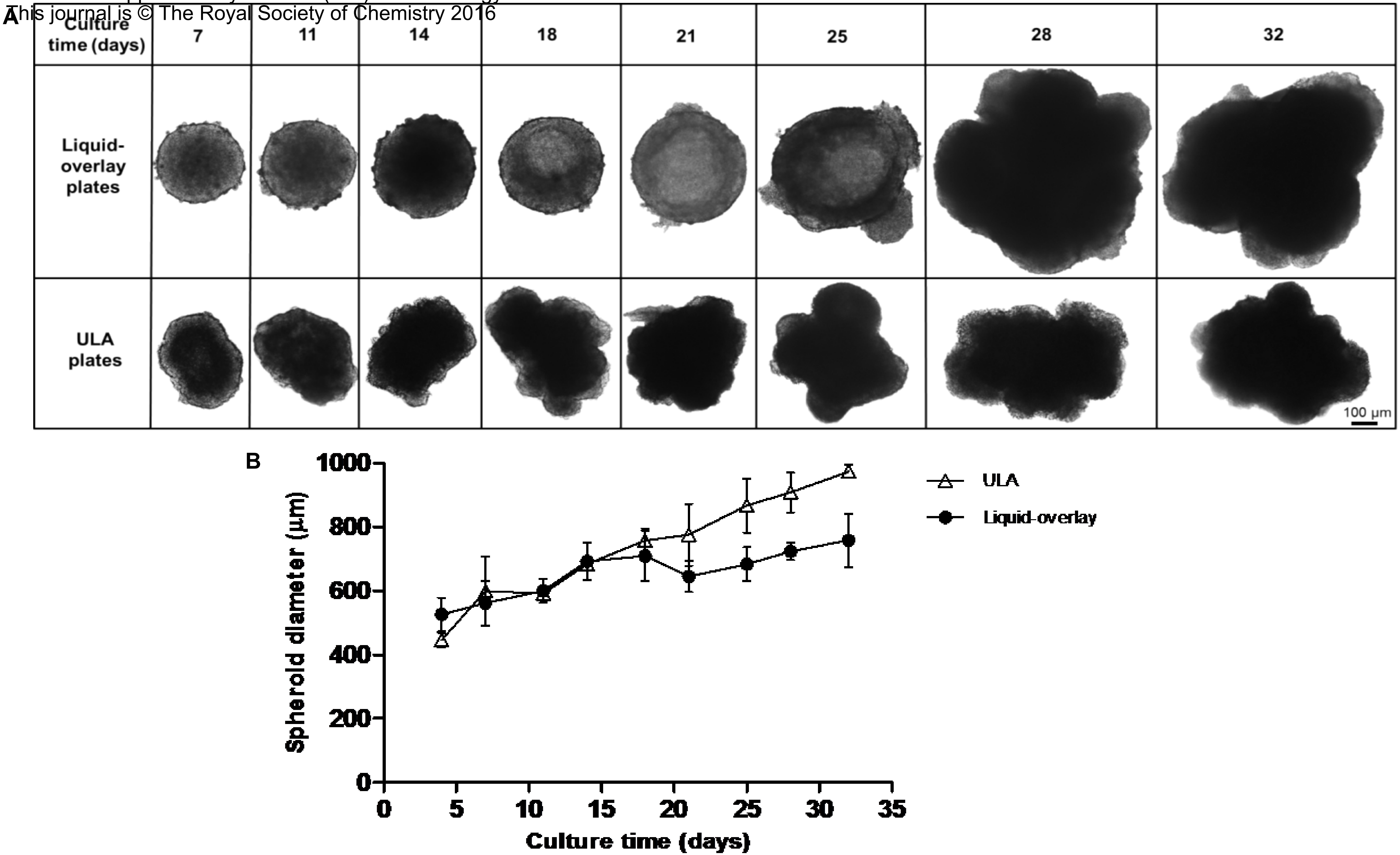

Supplementary Figure 1. Comparison of spheroid formation techniques. *(**A**) Phase-contrast images of spheroids cultured from 2500 cells on liquid-overlay or ULA plates over 32 days. Scale bar =100 µm. (**B**) Growth curve of spheroids cultured by liquid-overlay technique (black circle) or ULA plates (clear triangle). Spheroid diameter (µm) was plotted against culture time (days). Data are represented as mean ± standard error (n=3 in triplicate).*

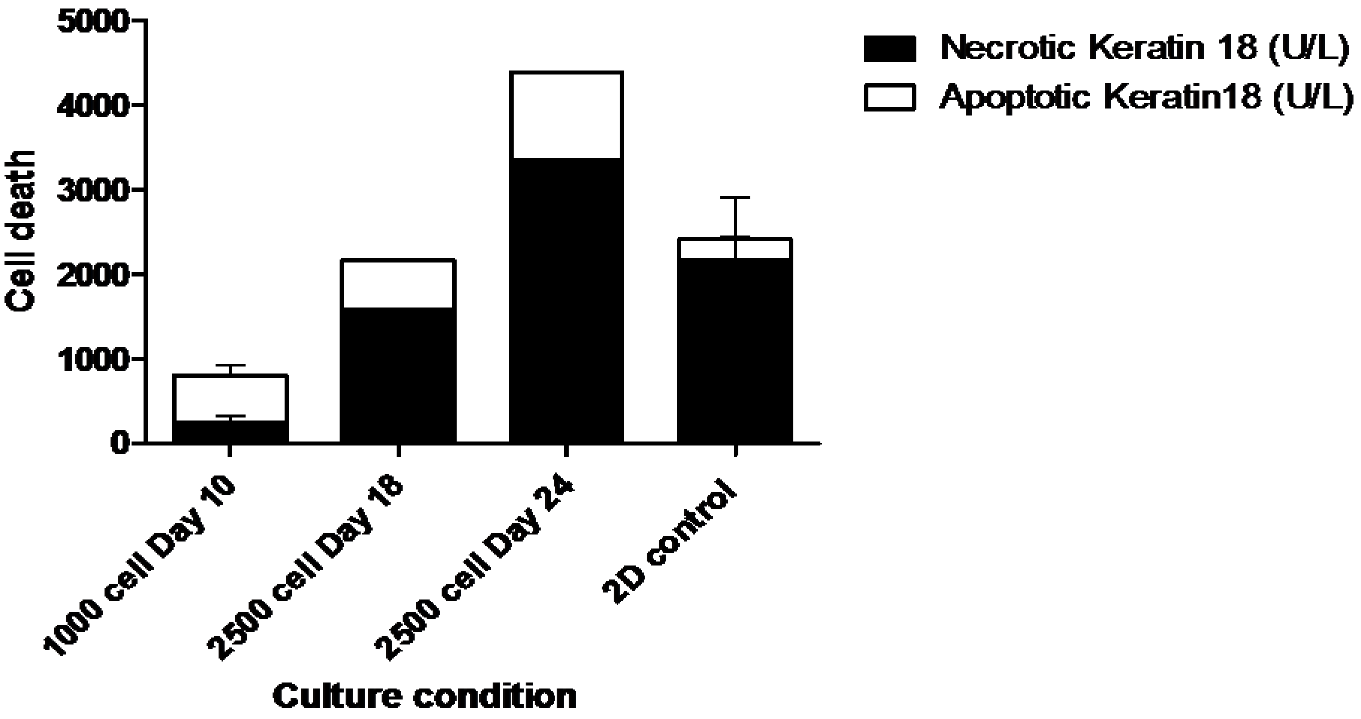

Supplementary Figure 2. Comparison of basal cell death in spheroids of different sizes. *Biomarkers of apoptosis (cleaved keratin 18) and total cell death (full-length keratin 18) were analysed in supernatants of spheroids of different sizes and culture times, and compared to a 2D monolayer control. Total cell death, split into apoptotic (white) and necrotic (black) are plotted against culture condition. Data are represented as mean ± standard error (n=3 using 20 replicates).*

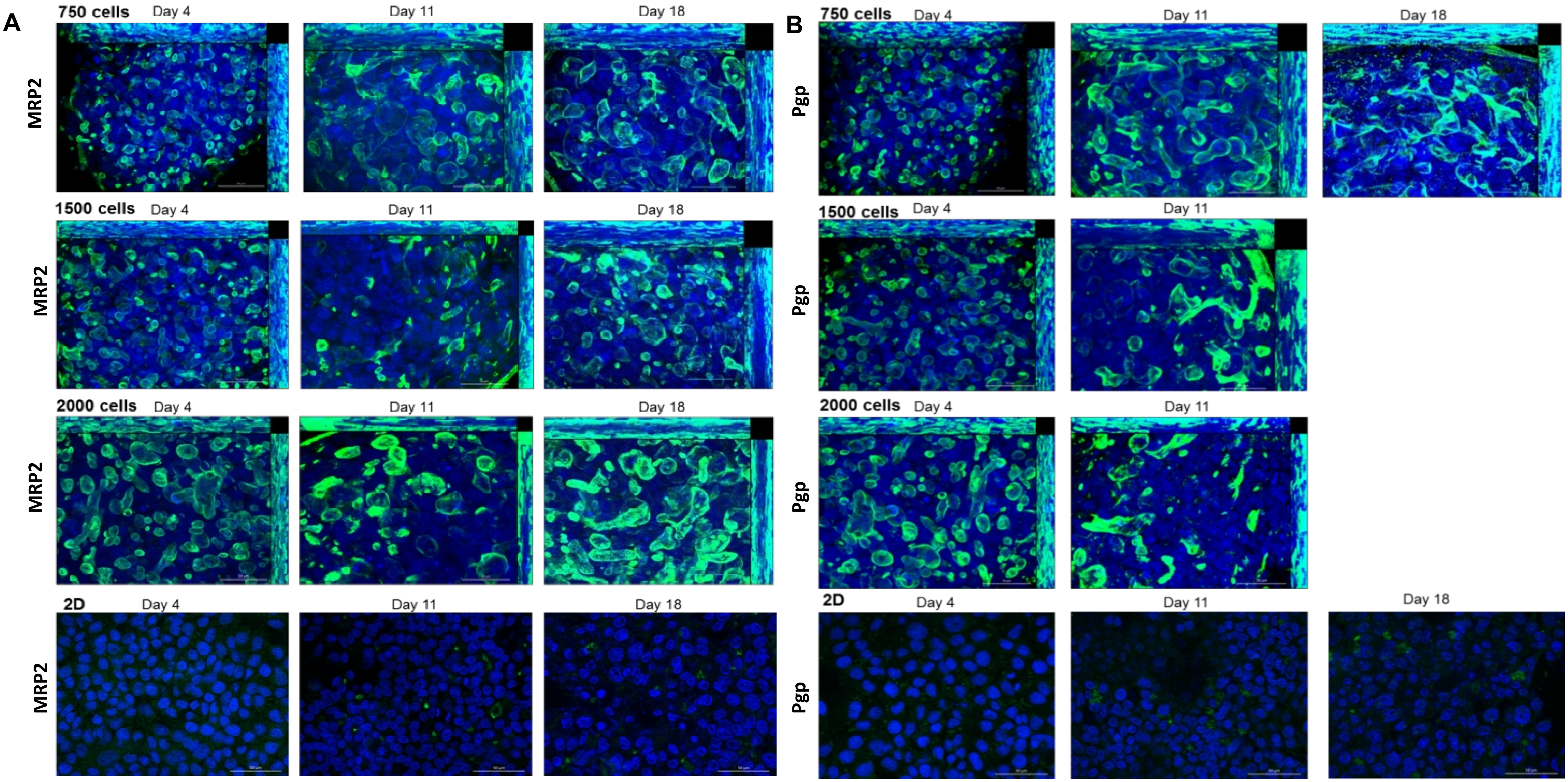

Supplementary Figure 3. Transporter polarisation in spheroids of different sizes. *Spheroids were created from 750, 1500 or 2000 C3A cells by liquid-overlay technique and compared to C3A cells cultured in a 2D monolayer. Samples were fixed at day 4, 11 and 18 of culture. Immunofluorescent staining was performed for the canalicular transporter (**A**) MRP2 (green) or; (**B**) Pgp (green) and Hoechst (blue) to view the nuclei. Maximum intensity projection images were taken using a Zeiss Axio Observer microscope. Scale bars = 50 µm.*

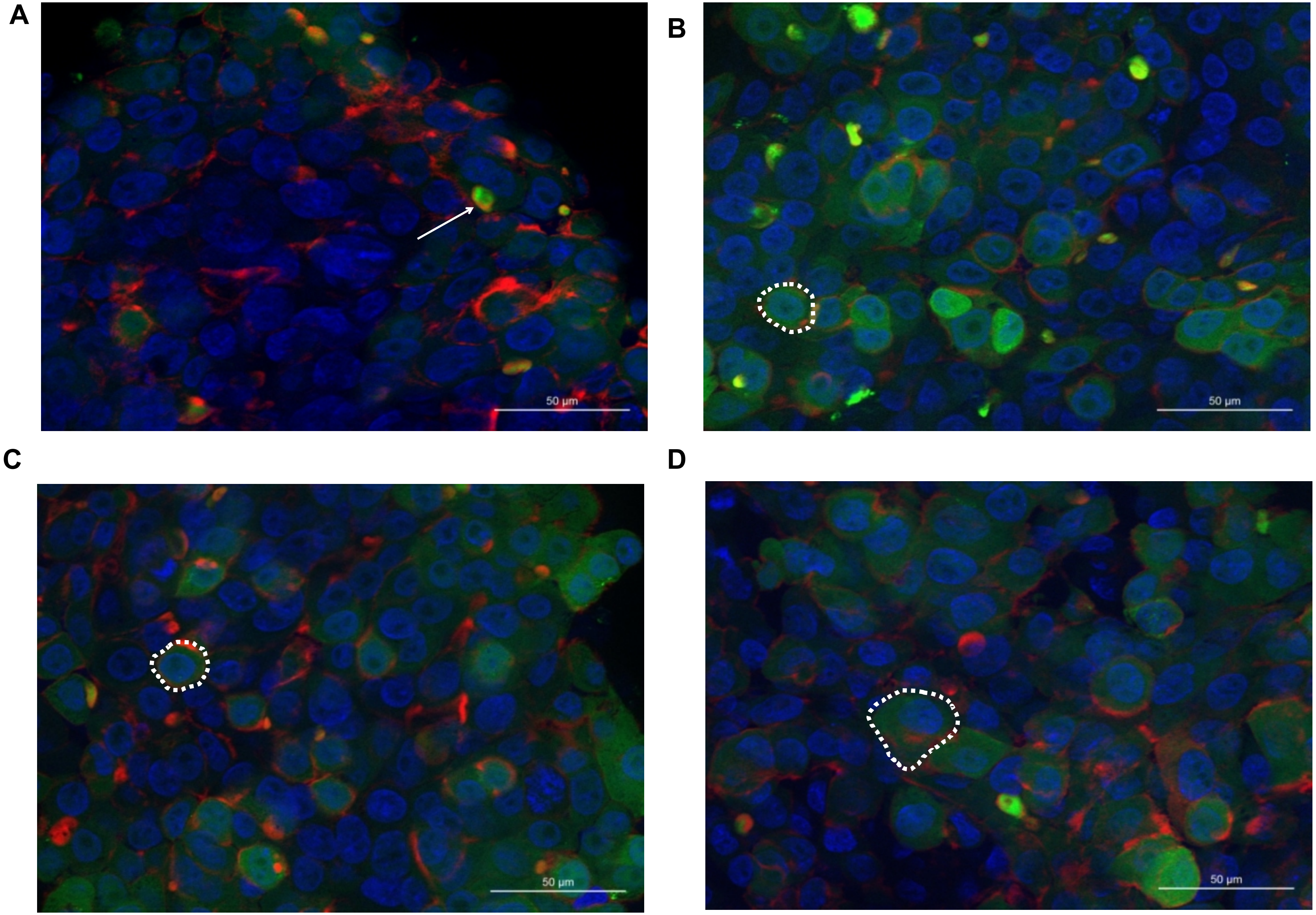

Supplementary Figure 4. Transporter function in spheroids. *Spheroids were created from 1000 C3A cells on liquid-overlay plates and then incubated with (**A**) CMFDA (green) only; (**B**) CMFDA and MK571 (MRP inhibitor); (**C**) CMFDA and PSC833 (Pgp inhibitor); (**D**) CMFDA, MK571 and PSC833 for 30 min. Spheroids were washed, fixed and stained with Hoechst (blue) to view the nuclei and phalloidin (red) to view F-actin. Images were taken using a Zeiss Axio Observer microscope. Dotted line indicates an example of a cell where CMDFA is retained within the cell cytoplasm. Arrow indicates the canalicular-like structures containing CMFDA. Scale bars = 50 µm.*

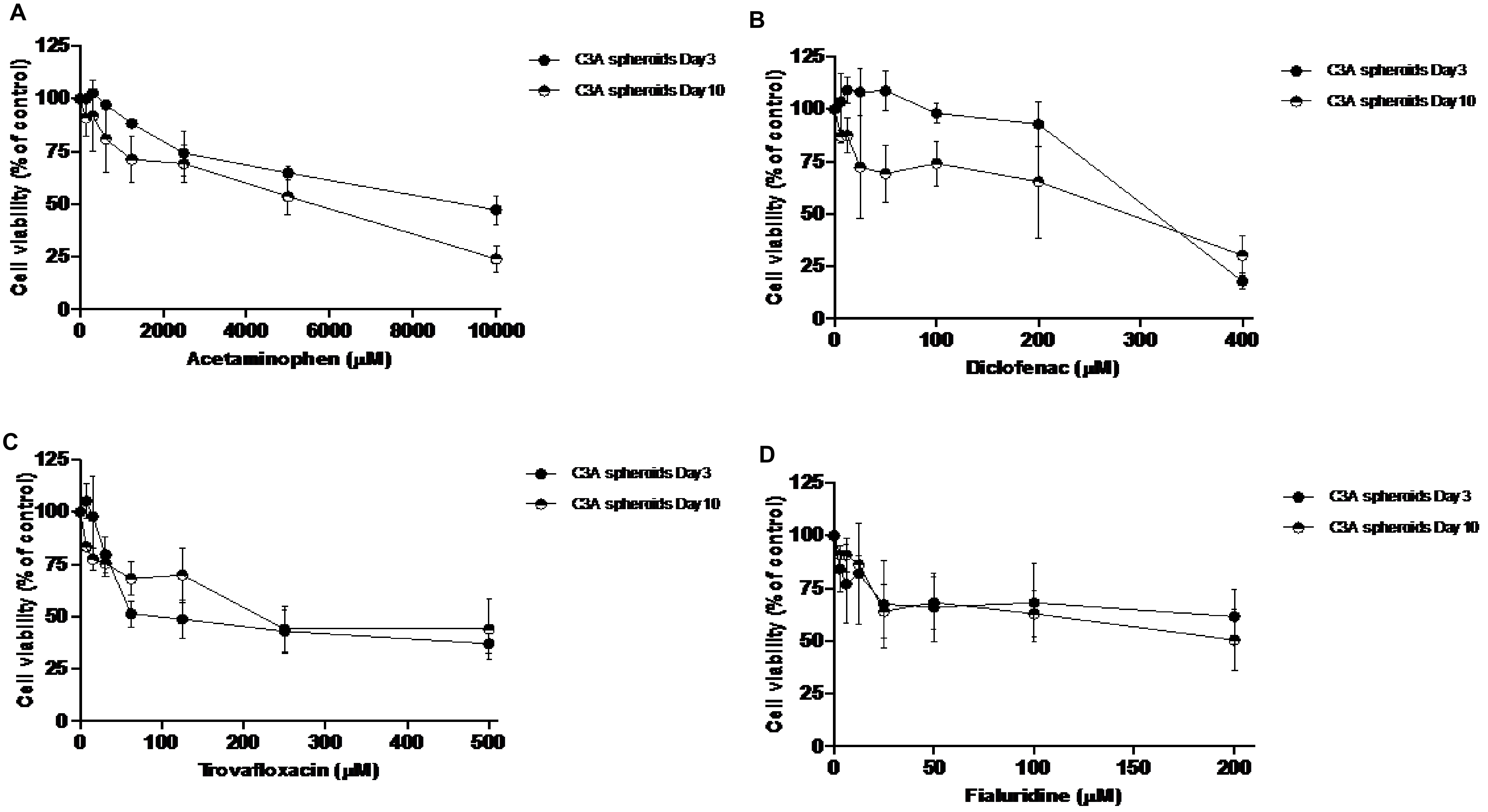

Supplementary Figure 5. Spheroids show a toxic response to hepatotoxins, regardless of culture time. *Spheroids were created from 1000 C3A cells by liquid-overlay technique and cultured for 3 days or 10 days, and treated with (**A**) acetaminophen; (**B**) diclofenac; (**C**) trovafloxacin and (**D**) fialuridine. Cell viability was analysed and plotted as a percentage of untreated control. Data are represented as mean ± standard error (n=3 in triplicate).*
